# A novel dry-bonding approach to reduce collagen degradation and optimize resin-dentin interfaces

**DOI:** 10.1038/s41598-018-34726-8

**Published:** 2018-11-15

**Authors:** Thiago Henrique Scarabello Stape, Roda Seseogullari-Dirihan, Leo Tjäderhane, Gabriel Abuna, Luís Roberto Marcondes Martins, Arzu Tezvergil-Mutluay

**Affiliations:** 1 Department of Restorative Dentistry and Cariology, Adhesive Dentistry Research Group, Institute of Dentistry, University of Turku, Turku, Finland; 2 Turku University Hospital, TYKS, University of Turku, Turku, Finland; 3 Department of Oral and Maxillofacial Diseases, University of Helsinki, Helsinki, Finland and Helsinki University Hospital, Helsinki, Finland; 4 Research Unit of Oral Health Sciences, Medical Research Center Oulu (MRC Oulu), Oulu University Hospital and University of Oulu, Oulu, Finland; 5 Piracicaba Dental School, University of Campinas, Department of Restorative Dentistry, Dental Materials Area, Piracicaba, SP Brazil; 6 Piracicaba Dental School, University of Campinas, Department of Restorative Dentistry, Piracicaba, SP Brazil

## Abstract

In dentistry, the wet-bonding approach relies on water to maintain demineralized collagen expanded for proper resin infiltration; nevertheless, hydrolytic instability of the resin-dentin interface is inevitable with current bonding techniques. Considering dimethyl sulfoxide’s (DMSO) ability to “biomodify” collagen and precipitate enzymes, the aim was to test whether the use of DMSO would permit adequate resin bonding to H_3_PO_4_-etched dehydrated dentin and assess its impact on collagen degradation by host-derived enzymes. Etched dentin surfaces from extracted sound human molars were randomly bonded in wet or dry conditions using aqueous or ethanolic DMSO solutions as pretreatments and bonding resins with or without DMSO. Bonded teeth were sectioned into resin-dentin slabs for confocal *in situ* zymography and beams for microtensile bond strength test. Demineralized powdered dentin was incubated in the tested DMSO -media and a hydroxyproline assay evaluated dissolution of collagen peptides. Zymography was performed on protein extracts obtained from dry and wet H_3_PO_4_-ecthed dentin powder treated with the DMSO- media. The correlative biochemical analysis demonstrated that reduction of water content during dentin hybridization by the innovative dry-bonding approaches with DMSO is effective to inactivate host-derived MMP-2 and MMP-9 and thus reduce collagen degradation while simultaneously optimizing resin-dentin bonding.

## Introduction

Resin-dentin bonding is a revolutionary form of *in situ* tissue engineering in which intrinsically hydrated demineralized collagen^1^ serves as a scaffold for resin infiltration to couple dental adhesives to the underlying mineralized dentin^2,3^. Nonetheless, limitations in this complex bonding process contribute to formation of imperfect hybrid layers invariably fated to failure after prolonged function^2–4^. Incomplete^5^ and suboptimal^6^ resin infiltration, the inability of current bonding resins to completely replace free and loosely bound water within collagen matrix^7^, hydrolytic instability of hydrophilic methacrylate monomers^8^ and collagen degradation^2,9^ remain as major drawbacks to the longevity of resin-dentin bonds^4^. Curiously, such processes can be considered as highly correlative since their resultant degradative effects occur exclusively in the presence of water^2^.

Water actively participates in resin-dentin bond degradation^10^ serving as a functional medium for the hydrolysis of resin matrices by esterases^11^ and collagen by both endogenous and exogenous collagenolytic and gelatinolytic enzymes (*i.e*. matrix metalloproteinases and cysteine cathepsins)^2,12^. Notably, especially the etch-and-rinse approach requires a partially wet substrate to maintain adequate interfibrillar spaces within demineralized collagen for proper resin infiltration^13^. There is a general consensus that the presence of unprotected water-filled fibrils^14^ creates a weak link highly prone to hydrolytic degradation^2–4,12^. Hence, the main questions reside on how to eliminate free and loosely bound water^1^ from demineralized collagen in clinically feasible time frames without jeopardizing resin-dentin bonding and whether the reduction of such water-filled zones would have an impact on collagen degradation.

Dimethyl sulfoxide (DMSO) is one of the most versatile solvents in biological sciences with a unique ability to “biomodify” demineralized collagen^15,16^, thus favoring resin-dentin bonding^17^. DMSO’s interaction with water^18^ and its capacity to bind, precipitate and unfold hydrophobic proteins^19^ may synergically contribute for this matter. The aim of this study was to examine the central hypothesis that binary solutions of DMSO, dissolved in either ethanol or water, and a DMSO-containing *primer* would permit adequate resin bonding to dehydrated demineralized dentin while reducing collagen degradation by host-derived enzymes. The tested null hypotheses were that: (i) dry-bonding using DMSO would have no impact on dentin bond strength; and (ii) the relative proteolytic activity of H_3_PO_4_ etched-dentin would not be affected by DMSO-pretreatments.

## Results

### Dentin bond strength

**Figure 1:**
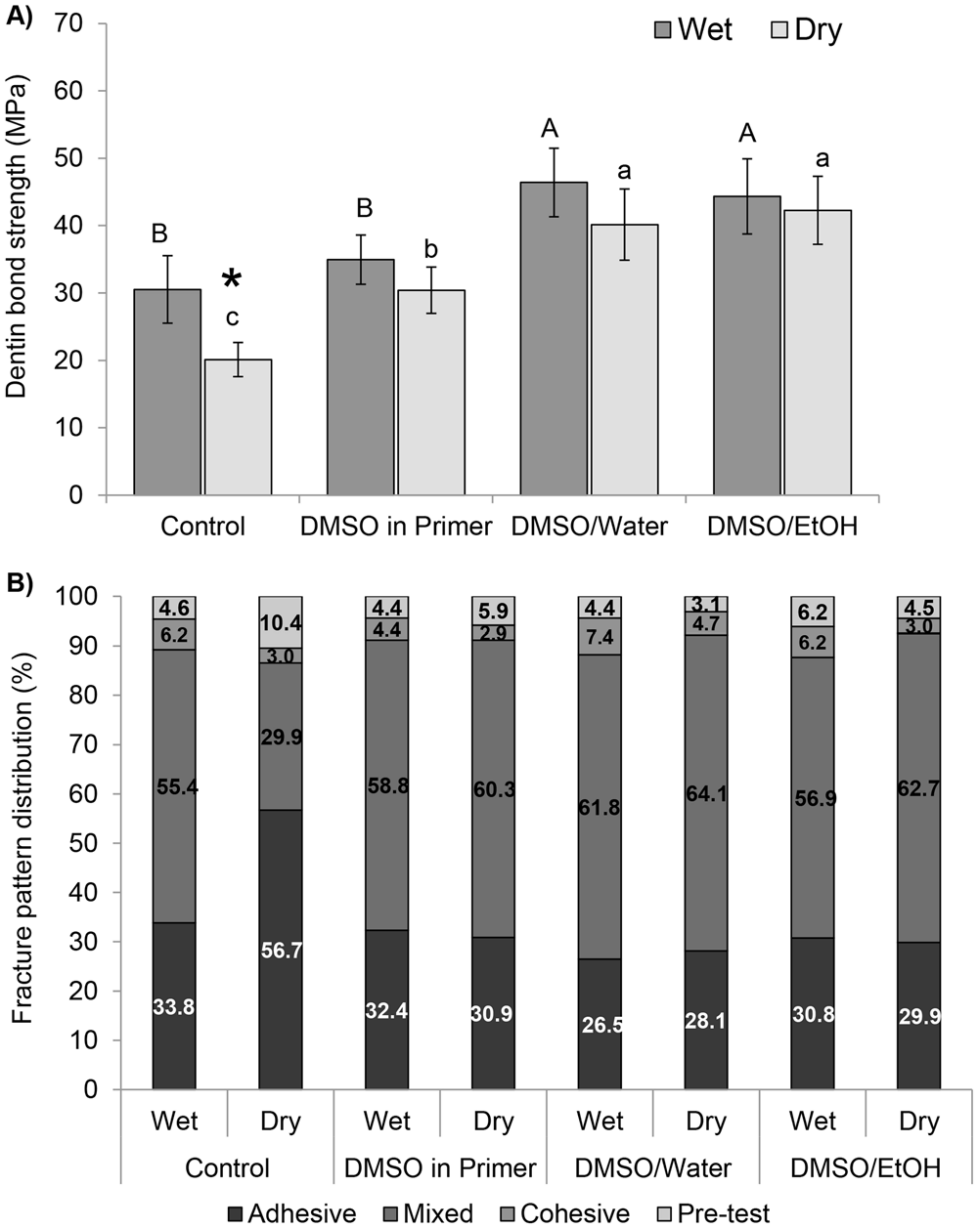
(**A**) Microtensile bond strength of wet- and extensively air-dried demineralized dentin bonded with SBMP using DMSO solutions as dentin pre-treatments or incorporated in the bonding agent (DMSO/SBMP). Pretreatment solutions consisted of DMSO dissolved in either water (DMSO/H2O) or ethanol (DMSO/EtOH). Heights of bars indicate the mean values (MPa) of 8 teeth per group (n = 8) and standard deviations. Columns identified by different capital letters represent significant differences according to Tukey Test (p < 0.05) for wet-dentin groups. Columns identified by different lowercase letters represent significant differences for dry-dentin groups. * represent significant differences between wet- and dry-dentin for each pre-treatment. (**B**) Graphical presentation of proportional prevalence of fracture modes for all experimental groups.

Two-way ANOVA revealed that “dentin condition” (*p* = 0.00004; *η*^2^_*p*_ = 0.318) and the “use of DMSO” (*p* < 0.0001; *η*^2^_*p*_ = 0.761) had significant effects on bond strength regardless their interaction (*p* = 0.0786). The mean cross-sectional area of tested resin-dentin beams (0.81 mm^2^ ± 0.2) ranged from 0.77 to 0.88 mm^2^ without significant statistical different regarding specimen size (*p* = 0.793). The bond strengths are reported in Fig. 1A. Dry-bonding caused a significant 33% reduction of bond strength (*p* < 0.05), while incorporation of DMSO into SBMP *primer* produced bond strength values similar to wet-bonding. Pretreatments with DMSO solvated in either water or ethanol significantly increased immediate bond strengths (*p* < 0.05) and were not statistically different from each other irrespective of dentin condition (*p* < 0.05). Fracture patterns (Fig. 1B) were mostly mixed for all groups with the exception of the control dry-bonded control group which presented a substantial 60% increase in adhesive failures compared to the conventional wet-bonding protocol.

### *In situ* zymography

**Figure 2:**
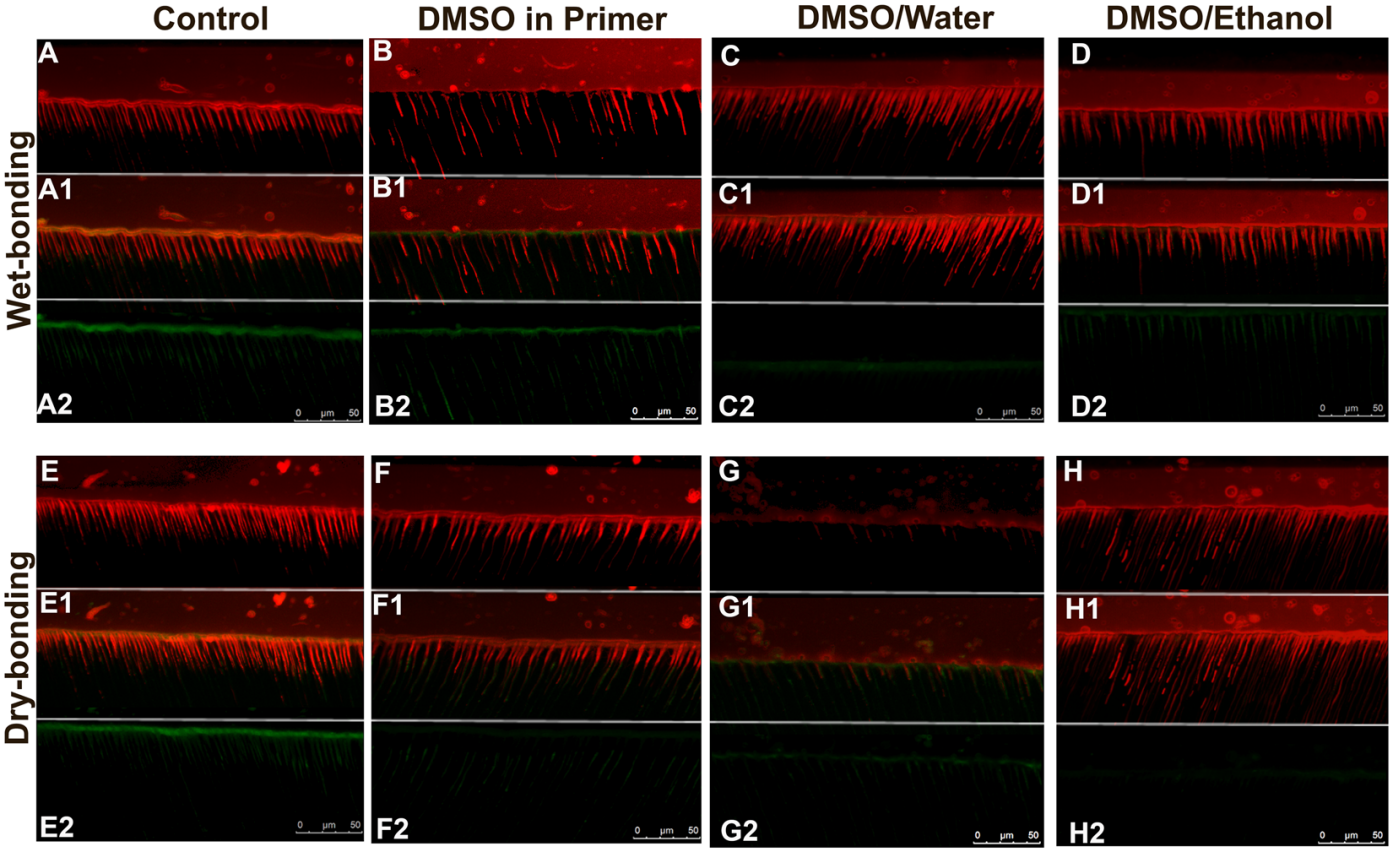
Representative CLSM scans (63x/1.4NA oil immersion objectives) for in situ zymography of wet- and extensively air-dried demineralized dentin bonded with SBMP. DMSO was solvated in water (DMSO/Water) or in ethanol (DMSO/Ethanol) and used as dentin pre-treatments or incorporated in the bonding agent (DMSO in Primer). Isolated red fluorescence signals, originated from Rhodamine B in adhesive, delineate the morphology of adhesive interface (**A**–**H**). Green fluorescence signals designate collagenolytic activity originated from quenched FITC-conjugated collagen breakdown by endogenous enzymes. (A1-H1) depict the localization of collagenolytic activity on the hybrid layer and surrounding areas. (A2-H2) show isolated FITC fluorescence revealing different levels of collagenolytic activity according to the different pretreatments and dentin conditions. While untreated control groups (A2 and E2) exhibited higher FTIC fluorescence signals, pretreatments with DMSO/H2O (C2 and G2) and DMSO/EtOH (D2 and H2) indicate reduced endogenous enzymatic activity especially on dry dentin (F2 and G2). Incorporation of DMSO in the bonding resin produced similar FITC fluorescence signals (D2) to control group in wet condition; however, a slight reduction of enzymatic activity in the hybrid layer was observed for the dry-bonding protocol (H2).

Representative CLSM images of the tested resin–dentin interfaces exhibiting hydrolysis of the FITC-conjugated collagen by the endogenous enzymatic activity are shown in Fig. 2. Signs of collagenolytic activity were detected in all samples at the hybrid layer, underlying intertubular dentin and inside dentinal tubules. Untreated groups presented substantial collagen breakdown in both wet and dry dentin (Fig. 2A2,E2). DMSO/H_2_O (Fig. 2C2,G2) and DMSO/EtOH (Fig. 2D2,H2) produced fewer areas with collagenolytic signals compared to wet and dry control groups, respectively. DMSO/EtOH on dry dentin presented the lowest levels of enzymatic activity of all groups (Fig. 2H2). Dentin condition influenced the collagenolytic activity of DMSO incorporated in the bonding resin and DMSO/EtOH producing slightly better inactivation levels in dry conditions than in wet groups, respectively. For wet dentin, incorporation of DMSO in the bonding resin (Fig. 2B2) produced fluorescence levels almost similar to control groups; however, a clear reduction was observed on dry dentin (Fig. 2F2).

### Collagen dissolution

**Figure 3:**
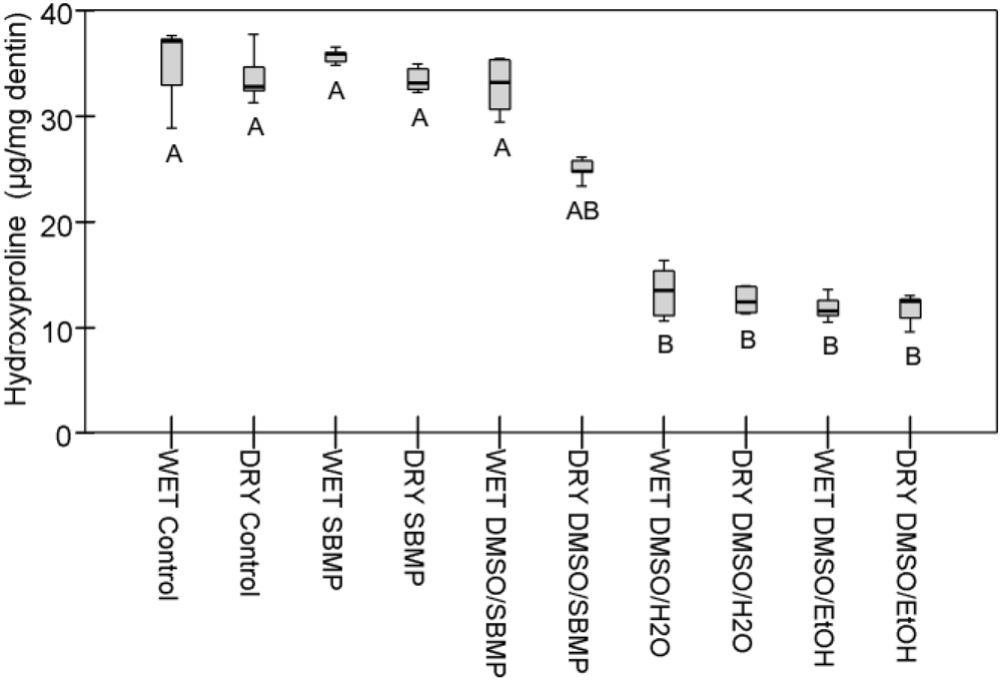
Hydroxyproline content derived from wet- and dry- demineralized dentin powder (n = 5) treated with DMSO after incubation for 7 days at 37 ºC. Control groups were incubated in distilled water (WET Control and DRY Control). SBMP also served as a control incubation solution (Wet SBMP and Dry SBMP). Experimental treatments consisted of DMSO solvated in water (DMSO/H2O), ethanol (DMSO/EtOH) or incorporated in the SBMP primer (DMSO/SBMP). Dissolved collagen from the demineralized dentin was expressed as μg hydroxyproline per mg dry mass of the baseline demineralized dentin powder. Groups with different upper ase letters were significantly different (p < 0.05) according to Dunn’s multiple comparison test.

The amount of hydroxyproline (µg/mg dry dentin) released from the demineralized dentin matrix is shown in Fig. 3. “Incubation solutions” had a significant effect on hydroxyproline release (*p* < 0.0001; Kruskal-Wallis). Hydroxyproline release from wet and dry demineralized dentin powder incubated in dH_2_O and SBMP *primer* were not statistically different (*p* < 0.05), showing that SBMP *per se* had no impact on collagen dissolution, irrespective of dentin condition. In contrast, both DMSO solutions significantly reduced collagen breakdown over 66% when compared to the controls (*p* < 0.05). No significant differences between the DMSO solutions were observed irrespective of dentin condition (*p* > 0.05). The DMSO-containing *primer* had a moderate reducing effect on hydroxyproline release without statistically significant differences to any other group (*p* > 0.05).

### Gel Zymography

**Figure 4:**
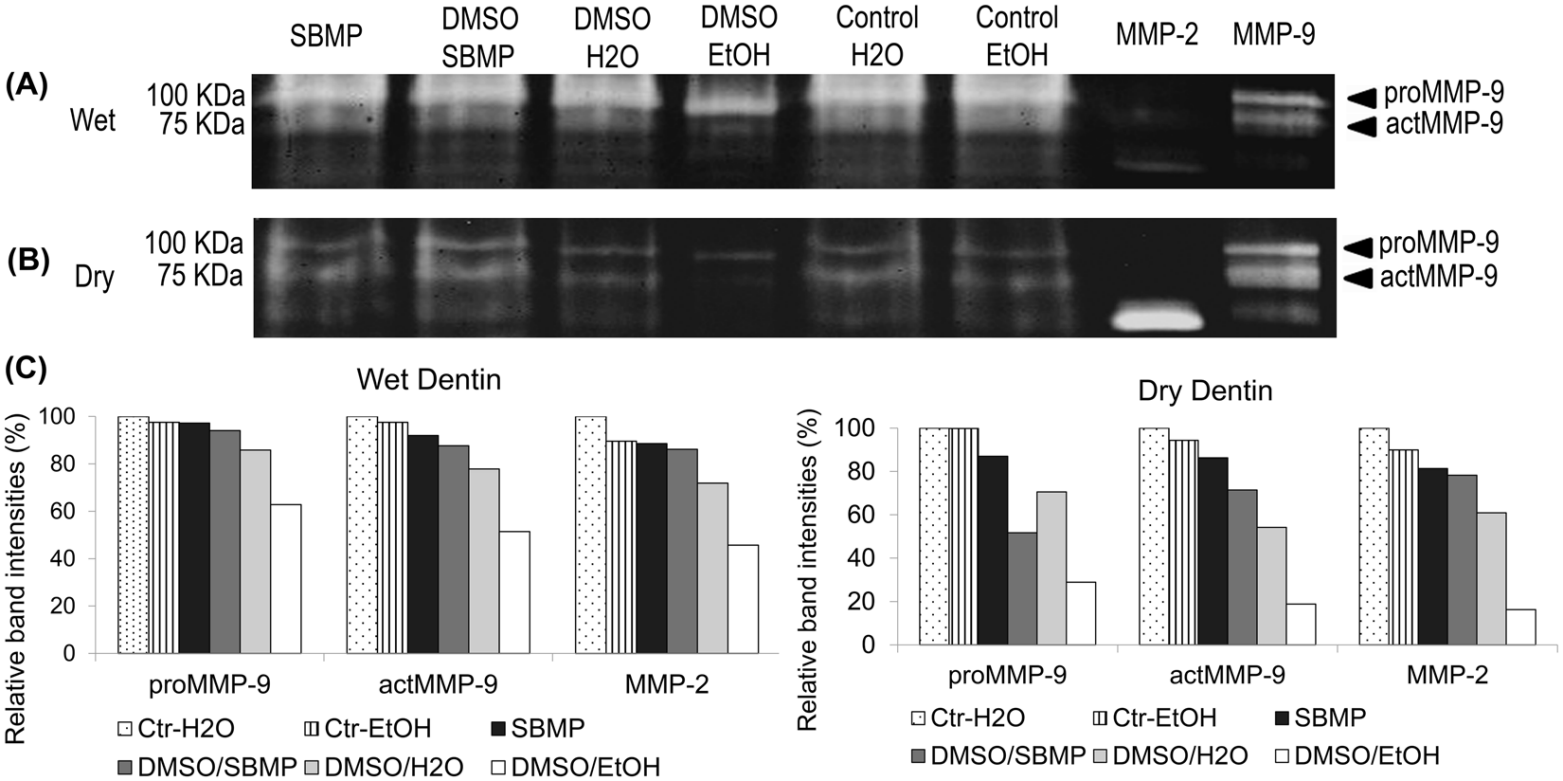
Gelatin zymograms of wet (**A**) and dry (**B**) demineralized dentin powder treated with DMSO solvated in water (DMSO/H2O), ethanol (DMSO/EtOH) or incorporated in SBMP. Control groups consisted of untreated dentin powder (Control H2O), SBMP and ethanol. Pure MMP-2 and MMP-9 extracts from odontoblasts were used as specific enzyme molecular mass standards. Molecular masses, expressed in kDa, are reported in the standard lane (Std). The graph (**C**) shows band intensities for proMMP-9, actMMP-9 and actMMP-2 calculated according to the peak area method. Complete inhibition of actMMP-2 and MMP-9 activity was not observed for neither of the DMSO treatments. Nevertheless, fainted bands indicate partial inactivation of MMP-2 and -9.

Zymograms of wet and dry demineralized dentin treatments are shown in Fig. 4A,B, respectively. Demineralized dentin exhibited pro- (92 kDa) and active (86 kDa) forms of MMP-9, MMP-2 in active form (66 kDa) and other minor bands with lower molecular weights (not shown). Analysis of band intensities using the peak area method (Fig. 2C) revealed a consistent partial inactivation of MMP-2 and −9 regarding the tested dentin conditions and treatments. Intensities of pro- and active MMP-9 and MMP-2 bands were similar for the wet dentin-treatments apart from DMSO/EtOH, which exhibited fainter active MMP-9 and MMP-2 bands compared to untreated demineralized dentin. A similar trend occurred for DMSO-treatments performed on dry-dentin, with the exception that DMSO/H_2_O presented fainter active MMP-9 and MMP-2 bands compared to control.

## Discussion

While the conventionally accepted wet-bonding approach requires residual water to maintain demineralized collagen expanded for resin infiltration, the proposed DMSO-protocols produced improved resin bonding to dry dentin. In fact, the DMSO-containing hydrophilic *primer* (*i.e*. 10 wt%) produced comparable bond strengths to wet-controls and dentin pretreatments with higher DMSO concentration (*i.e*. 50 vol%) improved immediate dentin bonding of dry-dentin beyond conventional wet-bonding. Therefore, the first null hypothesis was rejected. The rationale for testing a bonding resin with relatively low DMSO-content is that incorporation of high DMSO concentrations may hamper the mechanical properties of dimethacrylate bonding polymers^20^, which in turn could compromise the bonded interface. According to the hydrogen bonding force (δh: (J/cm^3^)^½^) of Hoy’s solubility parameters, 50% DMSO/H_2_O (δh 26.8) and 50% DMSO/EtOH (δh 16.6) acted as collagen re-expanding solutions due to their higher δh than air-dried collagen (δh 14.8). DMSO’s ability to “biomodify” collagen structure, increasing spaces between collagen microfibrils^16^ and improving dentin wettability^15^ support the improved bonding effectiveness even under dry-conditions. Since DMSO is able to break water self-associative tendency^21^, 10% DMSO incorporation into the water-containing SBMP *primer* certainly increased dried-collagen re-expansion rate producing comparable bond strengths to conventional wet-bonding. It is evident that this simplified use of DMSO or, to a better extent, its use as a dentin pretreatment reduced technique sensitivity of the etch-and-rinse approach^13^ concomitantly allowing water removal from the bonded interface by the proposed dry-bonding technique. Optimized bonding efficiency combined with reduced water-content during dentin hybridization could greatly contribute to clinical long-term durability; however, further studies are necessary to test such hypothesis.

This work brings up compelling evidence that DMSO may not only enhance resin-dentin interaction as previously reported^15,22^, but it may also incorporate a new protective factor to the resin-dentin bonded interface: DMSO-pretreatments considerably reduced dentin endogenous collagenolytic activity. *In situ* zymography showed no effect of dry-bonding on collagen breakdown indicating that removal of residual water by air-drying is insufficient to interfere on endogenous proteinase activity within bonded interfaces. However, DMSO-pretreatments performed on both wet and dry dentin produced lower levels of collagen degradation at the hybrid layer, even after incubation in artificial saliva. A similar trend was observed in the activity levels of MMP-2 and MMP-9 and overall collagen solubilization indicating that the presence of DMSO partially inactivated endogenous enzymatic activity. This led to rejection of the second null hypothesis for the relative proteolytic activity of etched-dentin was reduced by DMSO. Since the effect of DMSO on the collagenolitic activity within the hybrid layer was affected by dentin hydration levels especially for DMSO/EtOH and DMSO in the *primer*, DMSO’s strong interaction with remaining water likely played an important role on DMSO-enzyme interaction. Determining the exact mechanism in which DMSO reduced collagen breakdown is beyond the scope of this study. However, it is important to note that the markedly low solubility of proteins in polar solvents^23^ does not apply to DMSO, which promptly dissolves common proteins^24^. Therefore, we speculate that DMSO’s ability to solubilize and interact with proteins concentration-dependently^19^ supports the possibility of enzymatic debinding from the collagen matrix by the DMSO solutions^16^, which would explain the reduction of collagen breakdown. In fact, in high DMSO-water concentrations (*i.e*. 40–70%; depending on the protein structure and hydrophobicity), enzymes may have a stronger affinity to DMSO than pure water producing what is known as preferential DMSO interaction^19^.

This condition in which DMSO binds to protein hydrophobic moieties leading to protein unfolding and thus denaturation^19^ could explain the enzymatic inactivation findings on DMSO-treated dentin. Preferential binding of DMSO is affected by the bulk concentration of DMSO, substrate hydration and protein polarity^19^. Less polar proteins tend to bind more DMSO as the solvent concentration increases and as the substrate hydration diminishes^19^. Therefore, reduced water availability in dry-dentin pretreated with DMSO/EtOH most likely maximized DMSO binding to endogenous enzymes explaining the highest enzymatic inactivation levels for the *in situ* and gel zymography. Nonetheless, the specific interactions between DMSO and endogenous dentin enzymes must be further evaluated.

The *in situ* zymography results are in accordance with the hydroxyproline quantification where dentin pretreatments with 50 vl% DMSO reduced collagen solubilization more efficiently than the 10 wt% DMSO-containing primer. The exception was that dentin condition (*i.e*. wet vs. dry) had no impact on collagen solubilization after DMSO-treatments. Unlike the DMSO application time of one minute for the *in situ* zymography analyses, for hydroxyproline quantification demineralized dentin powder was incubated in the different DMSO-containing media for 7 days at 37 °C to allow collagen breakdown by the endogenous enzymes. The longer incubation period and larger volume of incubation medium kept under constant shaking certainly potentialized DMSO ability to debind endogenous proteases from the demineralized collagen, irrespective of water-content within collagen. From a clinical stand point, longer DMSO application times are unfeasible so the amount of residual water should be considered in order to enhance the inactivation of endogenous enzymes by DMSO pretreatments.

This study demonstrates the proof of concept that bonding current relatively hydrophilic resins to extensively air-dried demineralized dentin becomes viable when DMSO is used as a pretreatment or incorporated in the bonding resin. Apart from immediate resin-dentin bonding optimization, DMSO partially inactivates endogenous MMPs at the hybrid layer, thus reducing collagen solubilization of dry dentin. The ability to remove water from bonded interface and simultaneously reduce collagen breakdown in a clinically relevant time frame brings new possibilities to create resin-dentin interfaces with higher longevity.

## Methods

Extracted sound human third molars were obtained with informed consent from patients (age 18–21) under a protocol approved by the University of Oulu, Finland (#23-2003). Tooth collections were performed in accordance with relevant guidelines and regulations. Original clinical indications for tooth extractions were not related to the present study. After extraction, teeth were stored at 4 °C in 0.9% NaCl containing 0.02% NaN_3_ to prevent microbial growth and were used within 1 month.

### Bonding procedures

Specimen preparation followed the general guidance for testing of dental composite bonding effectiveness^25^. Teeth were coronally sectioned under water cooling to remove occlusal enamel and to expose flat midcoronal dentin surfaces followed by root removal 2 mm below the enamel-dentin level. Exposed midcoronal dentin surfaces were wet-polished with 320-grit SiC paper for 60 s to create standardized smear layers. Crown segments (n = 8/group) were randomly allocated to 8 groups following a study design with two factors: (i) “dentin condition” in two levels composed of dry- and wet-bonding protocols; and (ii) “DMSO treatment” in four levels consisting of no treatment, use of 50 vol% DMSO (Dimethyl Sulfoxide, Sigma-Aldrich, St Louis, MO, USA) dissolved in either water (DMSO/H_2_O) or ethanol (DMSO/EtOH), and incorporation of 10 wt% DMSO in the bonding resin (Adper Scotchbond Multi-Purpose: SBMP, 3 M ESPE, St Paul, MN, USA). In order to produce the DMSO-containing bonding resin, SBMP *primer* was evaporated at room temperature to remove 10 wt% of the original solvent composition and thus avoid changes in the original monomer-solvent ratio. Subsequently, 10 wt% DMSO was added to the evaporated aliquot followed by ultrasonic mixing for 60 s. Dentin surfaces were acid-etched with 32 wt% H_3_PO_4_ for 15 s (Scotchbond Universal Etchant, 3 M ESPE) and rinsed for 15 s with water. For the wet-bonding protocols, blot-drying with paper tissue was carefully performed leaving the dentin surface slightly moist. Conversely, dry-bonding was performed by continuous air blasts using a 3-way syringe at a distance of 10 cm for 30 s. After dentin etching and humidity control, dentin pretreatments were performed consisting of active application of 50 µL DMSO/H_2_O or DMSO/EtOH solutions on etched-dentin followed by blot drying until paper filters no longer absorbed liquids from the bonding surface by capillarity. SBMP, with or without DMSO, was applied totaling 20 s and light cured for 10 s. Composite build ups (Filtek Supreme, 3 M ESPE) were performed in two 2 mm increments and individually light-cured for 40 s. Light curing of all resin materials was performed using a LED device (Bluephase 20i, Ivoclare Vivadent, Schaan, Liechtenstein) delivering 1100 mW/cm^2^.

### Microtensile bond strength

The restored crown segments (n = 8/group) were stored in distilled water for 24 h to allow water sorption and postoperative polymerization of the adhesive and resin composite to take place. Samples were then longitudinally sectioned into bar-shaped resin-dentin beams with cross-sectional area of approximately 0.8 mm^2^ ^26^. Each specimen was individually fixed to a testing jig with cyanoacrylate glue and subjected to tensile load at a crosshead speed of 0.5 mm/min until failure (DL2000, EMIC, São José dos Pinhais, PR, Brazil). The force (N) required to fracture the sample and the dimensions of the cross-sectional area (mm^2^) were recorded with a digital caliber to the nearest 0.01 mm and the tensile bond strength (MPa) was calculated. The bond strength of a minimum of 8 resin-dentin beams was averaged to represent the bond strength of each tooth. Since the data was normally distributed (Shapiro-Wilk; *p* = 0.604) and homoscedastic (Levene test *p* = 0.321), they were analyzed by two-way ANOVA followed by Tukey test (α = 0.05). Failure modes were evaluated at 40× magnification under a stereomicroscope (Leica M60, Leica Microsystems) and classified as cohesive, adhesive, or mixed failures^17^.

### *In situ* zymography

*In situ* zymography was used to identify collagenolytic activity within the hybrid layer Two teeth per group were prepared for qualitative analyses of collagenolytic activity at the bonded interface^27^. Freshly reconstituted FITC-conjugated collagen (D-12060, Molecular Probes, Eugene, USA) was actively applied for 60 s on etched-dentin after DMSO-treatments were performed or previously to the application of the DMSO-incorporated SBMP *primer*. Bonding procedures were carried on for all groups as previously described except that SBMP *primer* and *adhesive* were doped with Rhodamine B powder 0.1 wt%. The samples were stored at 37 °C for 7 days in calcium- and zinc-containing artificial saliva (5 mM HEPES, 2.5 mM CaCl_2_·H2O, 0.05 mM ZnCl_2_, and 0.3 mM NaN_3_, pH 7.4), individually sectioned into a minimum of four 0.6 mm thick slabs per tooth, wet-polished with 600, 1200 and 2000 SiC paper and ultrasonically cleaned for 5 min after the polishing steps. The entire resin-dentin interface was examined using a multiphoton confocal laser microscope (CLSM: Leica SP5, Heidelberg, Germany) equipped with 63×/1.4NA oil immersion lens using a 488 nm argon laser (490–540 nm band pass filter) and a 563 nm laser (580–630 bandpass filter). The z-stack scans (0.5 µm) were compiled into single projections until 20 µm final volume. The emission of FITC signal allowed the identification of areas at the bonded interface presenting collagenolytic activity as a result of breakdown by endogenous enzymes. Sequential images of the bonded interface were recorded and qualitatively analyzed for the intensity and extension of FITC-conjugated collagen hydrolysis.

### Enzyme activity assays

Ninety extracted human third molars were ground free of enamel, the roots were sectioned off and pulp soft tissues were removed. Dentin fragments were frozen in liquid-nitrogen for 5 min followed by trituration at 24 Hz for 2 min in a ball-mill (Model MM400, Retsch, Newtown, PA, USA). Dentin powder was then sieved (Advantech Sonic Sifter, Advantech Mfg., New Berlin, MN, USA) to uniform particle size at <300 μm.

Collagen solubilization was assessed by hydroxyproline quantification. Five g of dentin powder were demineralized in 10 wt% H_3_PO_4_ (pH ≈ 0.4) for 10 min, centrifuged at 12000 rpm for 20 min at 4 °C and rinsed twice with 1 ml dH_2_O. Demineralized dentin powder was dehydrated in a silica desiccator for 72 h at 4 °C in order to remove loosely-bound water and divided (25 mg/sample) into 12 groups (n = 5). Half of the samples were rehydrated with 5 µL/sample dH_2_O. Wet and dry samples were incubated in a shaking bath for 7 days at 37 °C in 1 mL of tested pretreatment solutions (*i.e*. DMSO/H_2_O and DMSO/EtOH) and in the DMSO-containing *Primer* (DMSO/SBMP). Dry control samples were incubated in dH_2_O or SBMP *Primer*. At the end of the incubation, 25 μL of the media was collected from each vial, freeze-dried for 72 h (Alpha 1–5, Martin Christ Gefriertrocknungsanlagen, Osterode am Harz, Germany) for solvent removal, subsequently re-suspended in 75 μL of dH_2_O and transferred to individually labeled ampules. Solubilized collagen peptide fragments were assessed following a previously described hydroxyproline quantification protocol^28^. The specimens were re-suspended with 25 µl water after freeze-drying. Aliquots of standard hydroxyproline (2–20 µg) prepared from stock solutions and test samples containing hydroxyproline under 10 µg/ml were mixed with 25 µl of 4 N sodium hydroxide (2 N final concentration) in a total volume of 50 µl in 2 ml Nalgene O-ring tubes. The samples were hydrolyzed by autoclaving at 120 °C for 20 min. 450 µl of chloramine-T was added to hydrolyzed tubes and mixed gently to allow oxidation for 20 min at room temperature. 500 µl Ehrlich’s aldehyde reagent was added to each specimen for chromophore formation by incubating the specimens at 65 °C for 20 min. Absorbance values were obtained in a spectrophotometer (Model UV-A180, Shimadzu, Tokyo, Japan) at 550 nm and plotted against the standard hydroxyproline curves to determine the hydroxyproline release (μg/mg of dry dentin). Data was analyzed by Kruskal–Wallis one-way ANOVA on ranks and Dunn’s multiple comparison tests (α = 0.05).

A gel zymographic assay evaluated the effect of solvent and adhesive components on gelatinolytic activity of demineralized dentin extracts. Zymography was performed in accordance with Mazzoni *et al*.^29^. Briefly, demineralized dentin powder (200 mg/sample) was divided into 12 groups (n = 4) according to dentin condition (wet vs. dry) and treatment solutions (DMSO/H_2_O, DMSO/EtOH, DSMO/SBMP). dH_2_O, ethanol and SBMP *Primer* served as controls. For dry groups, demineralized dentin was dehydrated in a desiccator for 72 h. Dentin powder was treated with 400 μL of the DMSO solutions and vortexed for 60 s and centrifuged to remove the supernatant. Samples were re-suspended in 1.8 mL extraction buffer for 24 h at 4 °C under constant stirring, sonicated for 20 min and centrifuged at 12000 rpm for 20 min at 4 °C. Sample aliquots were concentrated using a centrifugal concentrator device (10,000-kDa cut-off, Vivaspin Sartorius Stedim Biotech, Goettingen, Germany) for 30 min at 20 °C (10,000 rpm) until the volume was reduced to 20 μL. The Bradford assay was performed to determine total protein concentrations. One hundred micrograms of protein aliquots were diluted in Laemmli sample buffer and subjected to electrophoresis under non-reducing conditions in 10% sodium dodecyl sulfate-polyacrylamide (SDS-PAGE) gel containing 1 mg/mL gelatin which had been fluorescently labeled with MDPF. A SDS-PAGE molecular weight standard (Dual Color Standards, Bio-Rad), was used along with purified MMP-2 and MMP-9^16^ to allow specific match of corresponding MMP bands. After electrophoresis, the gels were washed for 30 min twice in 2.5% Triton X-100 with agitation, and incubated in activation solution for 48 h at 37 °C. The gels were monitored with UV light (Gel Doc XR System, Bio-Rad) to reveal the gelatinolytic bands in triplicate samples. Band intensities were calculated according to the peak area method with digital image-analysis software (ImageJ, National Institute of Health, Bethesda, MD, USA).

## Electronic supplementary material

Dataset 1

## Data Availability

The datasets generated during the current study are available from the corresponding author on reasonable request.
