# Supplementary material for: A novel dry-bonding approach to reduce collagen degradation and optimize resin-dentin interfaces
Source: Sci Rep. 2018 Nov 15;8:16890. doi: 10.1038/s41598-018-34726-8 (PMC6237771; doi:10.1038/s41598-018-34726-8)
Supplement: Supplementary file 1 — Dataset 1 [file 41598_2018_34726_MOESM1_ESM.pdf]

A novel dry-bonding approach to reduce collagen degradation and optimize resin-dentin interfaces

Thiago Henrique Scarabello Stape, Roda Seseogullari-Dirihan, Leo Tjäderhane, Gabriel Abuna, Luís Roberto Marcondes Martins, Arzu Tezvergil-Mutluay

Supplementary Dataset

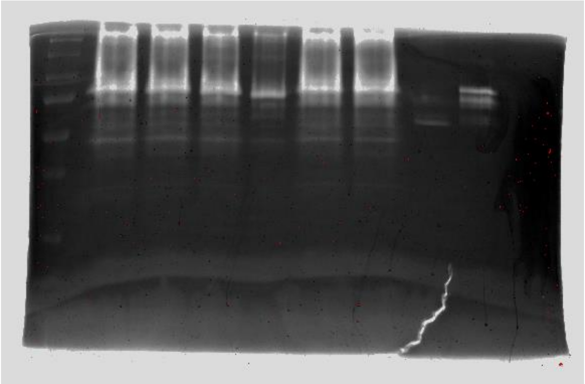

Figure 4 (A). Gelatin zymograms of wet demineralized dentin powder treated with DMSO solvated in water, ethanol or incorporated in SBMP. Control groups consisted of untreated dentin powder, SBMP and ethanol. Pure MMP-2 and MMP-9 extracts from odontoblasts were used as specific enzyme molecular mass standards.

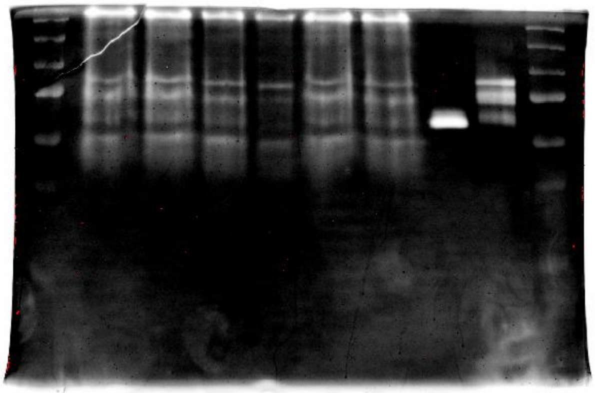

Figure 4 (B). Gelatin zymograms of dry demineralized dentin powder treated with DMSO solvated in water, ethanol or incorporated in SBMP. Control groups consisted of untreated dentin powder, SBMP and ethanol. Pure MMP-2 and MMP-9 extracts from odontoblasts were used as specific enzyme molecular mass standards.
